# Quadrat soil pollen signal reflects plant important values in forests and shrublands from subtropical China

**DOI:** 10.3389/fpls.2024.1348182

**Published:** 2024-03-20

**Authors:** Kai Li, Bin Tan, Mengna Liao, Jian Ni

**Affiliations:** ^1^ College of Life Sciences, Zhejiang Normal University, Jinhua, China; ^2^ Jinhua Mountain Observation and Research Station for Subtropical Forest Ecosystems, Jinhua, China

**Keywords:** plant community, plant important value, pollen analysis, quadrat investigation, subtropical China

## Abstract

Pollen analysis, a crucial tool in botany and ecology for examining historical biotic dynamics, has elicited debate owing to its complex link with vegetation. The challenge lies in discerning the ecological significance of pollen data. In this study, we conducted detailed quadrat surveys on Jinhua Mountain, subtropical China, analyzing topsoil pollen to determine whether pollen signals accurately reflect key ecological components in the forests and shrublands. We performed direct comparisons between pollen and plant compositions and calculated pollen percentages and plant Important Values (IVs) for each quadrat. The results indicate greater homogeneity in pollen composition across the study area compared to plant composition, particularly in the high percentage of *Pinus* pollen. However, distinct plant communities exhibited significantly different pollen compositions, as evidenced by the multi-response permutation test. This divergence aligns with variations in the dominant plant species across different communities. There were significant correlations between pollen percentages and plant IVs, with correlation coefficients of 0.55 (*p* < 0.001) at the quadrat level and 0.78 (*p* < 0.001) at the taxon level. These results support the utility of pollen analysis for representing ecologically significant values in subtropical Chinese forests and shrublands. Such correlations might also be extrapolated to pollen-based paleoecological studies.

## 1 Introduction

Pollen serves as a robust indicator for discerning spatial and temporal variations in species assemblages and interspecific interactions, rendering it well-suited for high-throughput evaluation of global ecological shifts (Hornick et al., 2021). Pollen analysis remains the predominant method for recovering historical data regarding vegetation, climate, and the environment (Prentice et al., 1998; Xu et al., 2007; Zhao et al., 2015; Birks, 2019; Chevalier et al., 2020; Zheng et al., 2021). The majority of investigations in this domain hinge upon the ecological significance of pollen, specifically its representation of vegetation (Davis, 1963; Parsons and Prentice, 1981; Sugita, 1994).

Understanding the complex relationship between pollen and vegetation is a fundamental aspect of pollen analysis. Influenced by various processes, including pollen production, transport, and deposition, this relationship is complex (Xu et al., 2007; Xu et al., 2016). To elucidate the pollen-plant connection and enhance the pollen-vegetation calibration, several approaches have been introduced and applied, including pollen representation value (*R* value), extended pollen representation value (*ERV*), relative pollen productivity (*RPP*), and relevant source area of pollen (*RSAP*) estimation (Davis, 1963; Andersen, 1970; Prentice and Parsons, 1983; Prentice, 1985; Prentice and Webb, 1986; Jackson and Kearsley, 1998; Sugita, 2007a, b). With the increasing accessibility of data related to pollen *R* values and *RPP* for pollen-vegetation calibration (Li et al., 2018; Wieczorek and Herzschuh, 2020; Xia and Ni, 2024), the pollen-vegetation relationship has gained substantial recognition. Chinese palynologists have also been dedicated to investigating this relationship, including *R* value, *RPP* estimation, and quantitative aspects of the pollen-plant connection (Yu et al., 2001; Xiao et al., 2011; Zheng et al., 2015; Xu et al., 2016; Li et al., 2017; Cui et al., 2020). Although uncertainties persist, regression correlations tend toward a linear curve in specific regions following calibration with *R* values and *RPP* (Xu et al., 2016). However, limited attention has been directed toward whether pollen can adequately reflect the role of a plant within a community. This represents a minor barrier between community ecology rooted in investigative approaches and pollen-based paleoecology (Seddon et al., 2014).

Pollen percentage constitutes the most commonly utilized data type in calculating pollen representation, *RPP*, and pollen source areas, whereas the type of plant data employed varies across different studies. For example, Andersen (1970) considered plant frequency, tree crown area, or tree basal area within a given plot, whereas subsequent forest-related studies frequently incorporated tree percentage (abundance) (Prentice and Webb, 1986; Jackson and Kearsley, 1998). In the context of steppe vegetation, coverage has been the prevailing metric for studying pollen-vegetation relationships (Xu et al., 2007). This choice is justified because counting individual plants is more challenging than estimating coverage. From a community ecology perspective, both abundance and coverage are crucial, as they provide quantitative insights into individual plants and the spatial extent they occupy (Song, 2016). However, abundance and/or coverage sometimes fall short of depicting a taxon’s functional role within the community. Instead, dominants or edificators play a pivotal role in shaping the community structure and maintaining its stability and ecosystem services. In vegetation ecology, dominants or edificators typically exhibit high importance values, amalgamating several measurements such as abundance, frequency, dominance, and height (Curtis and Mclntosh, 1951). Whether the pollen percentage aligns with numeric measures of taxon importance remains an open question.

To address these issues, we conducted extensive fieldwork on Jinhua Mountain, located in eastern subtropical China. Furthermore, pollen analysis was performed on the soil samples obtained from the quadrats. A vegetation map of Jinhua Mountain was compiled in 2017. Over the course of 2017-2018, plant quadrats from different vegetation types and plant communities were investigated to study the quantitively relationship between soil pollen percentage and plant community. Soil samples collected before the investigation were subjected to pollen identification. Our primary objectives were to compare discrepancies between pollen signals and the contemporary vegetation community in Jinhua Mountain and to determine whether pollen signals could effectively reflect significant ecological components within the subtropical Chinese plant community.

## 2 Materials and methods

### 2.1 Quadrat investigation and soil sample collection

Jinhua Mountain (E 119.48°–119.77°, N 29.15°–29.32°, 1312 m above sea level) is situated to the north of Jinhua City in Zhejiang Province in the middle-eastern region of China. This mountain predominantly consists of rhyolite with localized limestone occurrences. Influenced by a subtropical monsoonal climate, the mean annual temperature in the Jinhua region stands at 17.7°C. Notably, January recorded an average temperature of 5.2°C, while July experienced an average temperature of 29.5°C. The mean annual precipitation is 1436 mm, with 70% of the precipitation occurring between March and August. The vegetation on Jinhua Mountain is characterized by evergreen broad-leaved forests located at the northern boundary of the central subtropical zone. The region has abundant plant germplasm resources and a diverse array of plant species (Fan et al., 2019). As elevation increases, the vegetation transitions through various types, including evergreen broadleaf forests (*Castanopsis sclerophylla* (Lindl.) Schottky, *Schima superba* Gardner & Champ), evergreen deciduous broadleaved forests (*Liquidambar formosana* Hance, *Eurya muricata* Dunn, *Castanea seguinii* Dode), coniferous broadleaf mixed forests (*Pinus massoniana* Lamb., *Castanea henryi* (Skan) Rehder & E. H. Wilson, *Dalbergia hupeana* Hance), coniferous forests (*Pinus taiwanensis* Hayata, *Pin. massoniana*), and ultimately culminate in mountaintop shrublands dominated by *Rhododendron simsii* Planch. and *Rhododendron molle* (Blume) G. Don. Additionally, cultivated cedar (*Cunninghamia lanceolata* (Lamb.) Hook.) and hairy bamboo (*Phyllostachys edulis* (Carrière) J. Houz.) forests are also present in this region.

**Figure 1:**
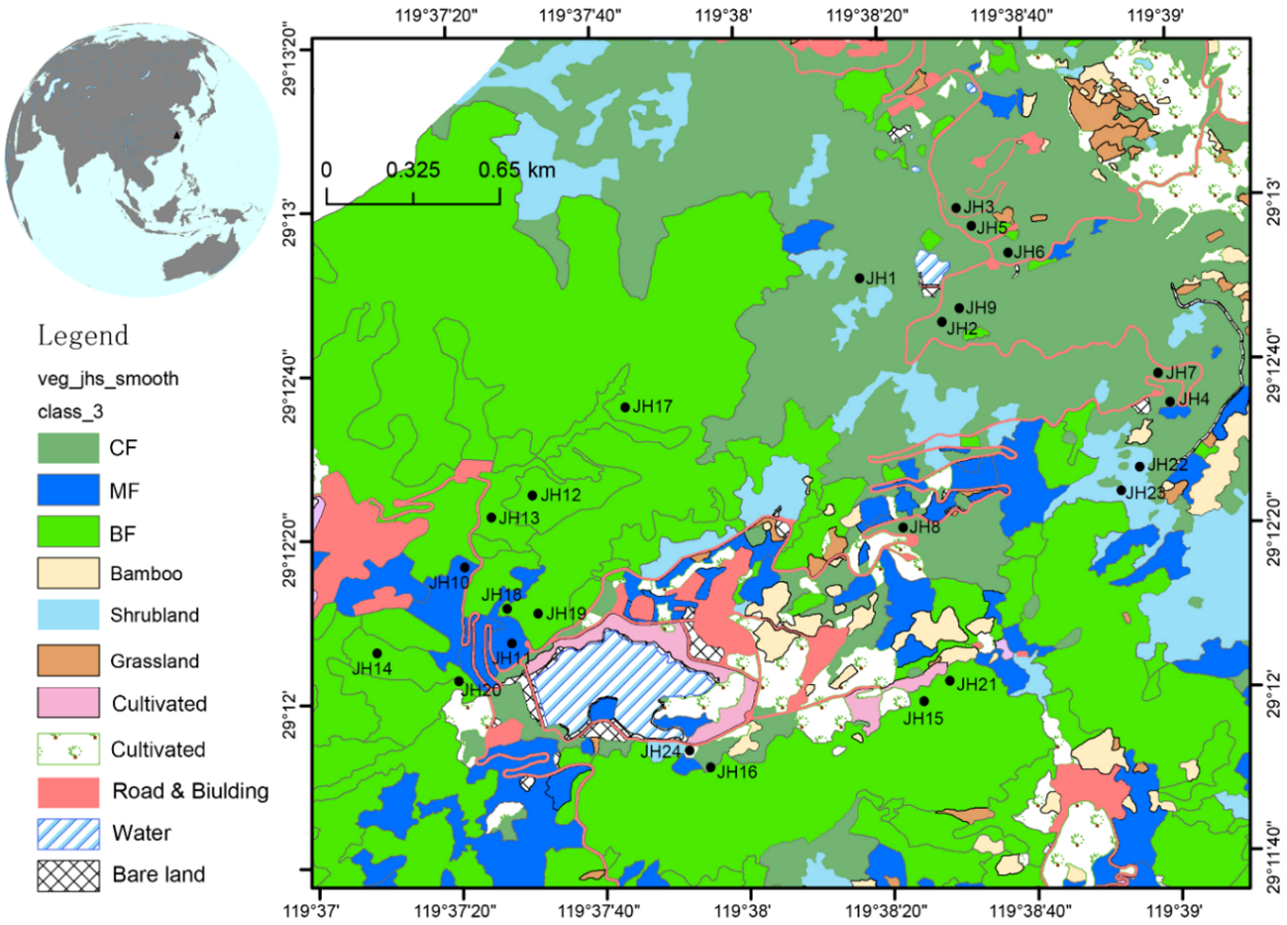
Map showing locations of Jinhua Mountain (top-left) and 24 quadrats in Jinhua Mountain (JH1-24). Vegetation map was cited from Fan (2020). CF, subtropical coniferous forest. MF, mixed coniferous and broad-leaved forest. BF, broad-leaved forest.

A comprehensive vegetation survey was conducted during 2017 and 2018. In total, 24 quadrats were rigorously selected, including five distinct vegetation types: coniferous forest, evergreen broad-leaved forest, mixed evergreen and deciduous broad-leaved forest, mixed coniferous and broad-leaved forest, and shrubland (**Figure 1**; **Table 1**). The dimensions of the quadrats were 30 × 30 m for forested areas and 30 × 10 m for shrubland regions. Each forested quadrat was further subdivided into nine 10 × 10 m sub-quadrats. Within these sub-quadrats, five 4 × 4 m plots were designated in the upper-left corner to scrutinize the shrub layer. Similarly, within the upper-left corner of each 4 × 4 m sub-quadrat, a 1 × 1 m quadrat was established to examine the herbaceous layer.

**Table 1:** Quadrat information in the Jinhua Mountain.

IDs	Latitude	Longitude	Altitude	Area (m)	Vegetation type	Canopy /Tree	Canopy /Shrub	Density (/m^2^)
JH1	119.64	29.21	1078	30 × 30	CF	0.8	0.6	0.71
JH2	119.64	29.21	1081	30 × 30	CF	0.8	0.5	0.49
JH3	119.64	29.22	1123	30 × 30	CF	0.65	0.5	0.22
JH4	119.65	29.21	961	30 × 30	CF	0.9	0.15	0.14
JH5	119.64	29.22	1110	30 × 30	CF	0.9	0.4	0.14
JH6	119.64	29.22	1100	30 × 30	CF	0.9	0.05	0.15
JH7	119.65	29.21	956	30 × 30	CF	0.9	0.3	0.26
JH8	119.64	29.21	711	30 × 30	CF	0.85	0.65	0.41
JH9	119.64	29.21	1091	30 × 30	CF	0.9	0.25	0.30
JH10	119.62	29.21	498	30 × 30	MF	0.8	0.7	0.68
JH11	119.63	29.2	567	30 × 30	MF	0.9	0.65	0.74
JH12	119.63	29.21	658	30 × 30	MF	0.85	0.5	0.68
JH13	119.62	29.21	550	30 × 30	MF	0.9	0.8	0.82
JH14	119.62	29.2	484	30 × 30	MF	0.85	0.6	0.82
JH15	119.64	29.2	598	30 × 30	BF	0.9	0.45	0.58
JH16	119.63	29.2	561	30 × 30	BF	0.9	0.6	0.64
JH17	119.63	29.21	644	30 × 30	BF	0.85	0.45	0.54
JH18	119.63	29.2	597	30 × 30	BF	0.9	0.6	0.57
JH19	119.63	29.2	608	30 × 30	BF	0.9	0.6	0.57
JH20	119.62	29.2	520	30 × 30	BF	0.9	0.65	0.79
JH21	119.64	29.2	541	30 × 30	BF	0.9	0.6	0.68
JH22	119.65	29.21	970	20 × 10	S	0.8	0.5	3.69
JH23	119.65	29.21	955	20 × 10	S	0.75	0.45	4.04
JH24	119.63	29.2	540	30 × 10	S	0.75	0.9	9.03

JH1, quadrat 1 in Jinhua Mountain. Vegetation type: CF, subtropical coniferous forest. MF, mixed coniferous and broad-leaved forest. BF, evergreen broad-leaved forest. S, shrubland.

All standing woody species with a diameter at breast height (DBH) equal to or exceeding 1 cm underwent rigorous examination, and their species name, plant count, DBH, height, crown width, and coordinates were duly recorded. For the shrub and herbaceous layers, species name, plant count, height, and coverage were documented. Within the shrubland quadrats, the species name, plant count, DBH, height, crown width, and coordinates of woody plants with a DBH of ≥ 1 cm were rigorously measured and recorded. For woody plants with a DBH < 1 cm, the species name, plant count, base diameter, height, and coordinates were documented for all trees with a height ≥ 10 cm.

Furthermore, within each 10 × 10 m sub-quadrat, a 1 × 1 m herbaceous quadrat located in the upper-left corner was designated to record the species name, plant count, height, and coverage within the herbaceous layer. Notably, at the commencement of the quadrat investigation, surface soil samples from to 0-1 cm depth were methodically collected within each 10 × 10 m sub-quadrat.

### 2.2 Laboratory works

The soil samples designated for pollen analysis underwent a series of rigorous treatments. Initially, they were subjected to heavy liquid flotation, which included treatment with HCl (10%) and KOH (10%) to eliminate carbonate and humic acid residues, respectively. Subsequently, the samples were passed through a 200 μm sieve and subjected to centrifugation. To process the samples further, we introduced 30 mL of ZnBr_2_ and performed centrifugation twice as a part of this procedure. Subsequently, the samples underwent two rounds of centrifugation with deionized water to remove the heavy liquid.

The final step of the process involved adding 5 mL of mixed acetic anhydride and concentrated sulfuric acid (9:1) to the samples, which were subsequently heated in a water bath at 80°C for 3 min. Subsequently, the samples were centrifuged and washed until the solution reached a neutral pH. To facilitate the quantification of pollen concentrations, tablets containing a known quantity of *Lycopodium* L. spores were introduced into each sample prior to the aforementioned treatments. Pollen was identified using a Zeiss Scope A1 at Zhejiang Normal University, with a minimum count of 500 terrestrial pollen grains per sample.

### 2.3 Numeric analysis

Numeric characteristics of vegetation communities, including abundance, coverage, frequency, and important values (IVs), are widely employed as valuable metrics in the field of community ecology (Song, 2016). IVs, in particular, serve as a crucial and commonly used index that reflects the comprehensive significance of individual taxa within a plant community (Curtis and Mclntosh, 1951). We computed the average IVs for each taxon based on the relative frequency, height, and dominance derived from the quadrat data.

Frequency was calculated as the number of plots in which a species was observed divided by the total number of survey plots. Relative frequency was computed by dividing the frequency by the sum of the frequencies of all species. Height was determined as the total height of individuals of a species, whereas the relative height was obtained by dividing the height by the sum of the heights of all species. Dominance was quantified as the total breast area of a species, and relative dominance was established by dividing dominance by the sum of the dominance of all species. Given the substantial importance of woody taxa compared to fern and herbaceous taxa in forest and shrub quadrats, we focused solely on calculating the IVs for woody taxa investigated within forest quadrats and shrub sub-quadrats. To facilitate comparisons with pollen results from the same quadrats, we summed the IVs of taxa with the same genus or family, aligning with the taxon names in the Chinese Pollen Database (Chen et al., 2021).

The percentage of pollen was initially computed for each surface soil sample. The percentage of woody taxa was derived from the total tree and shrub pollen counts, whereas the herbaceous and fern taxa were based on the overall pollen counts. The pollen percentage was subsequently calculated as the average percentage within each quadrat for the numerical analysis. To create vegetation and pollen stratigraphic diagrams, we utilized the “rioja” package in R (Juggins, 2022).

For further analysis, we selected the IVs of woody taxa from the quadrat investigation and the percentage of woody taxa from the pollen identification. Both datasets underwent square-root transformation to stabilize variances and optimize the “signal” to “noise” ratio. To test the dissimilarity originating from different vegetation types, we employed the Multi-response Permutation Procedure (MRPP) utilizing the Bray-Curtis coefficient as a dissimilarity measure (Warton et al., 2012). To assess the consistency of taxa between plant IVs and pollen signals, we conducted ordination-based Procrustes rotation analysis. Initially, all taxa present in both the vegetation and pollen datasets were included in the ordination analysis to compare site consistency. Subsequently, only taxa appearing in both datasets were considered in the data subsets to evaluate the differing contributions of shared taxa.

Detrended Correspondence Analysis was initially applied, with the first axis lengths for the IVs and pollen datasets measuring 3.43 and 1.42, respectively. Consequently, Principal Component Analysis (PCA) was performed. The first two PCA axes were explored for both vegetation and pollen data. To measure the similarity between vegetation and pollen, we performed Procrustes rotation analysis using a standard method of multidimensional scaling on the first four PCA axes (Peres-Neto and Jackson, 2001). The significance of the similarity between the two datasets was assessed using the protest procedure. All analyses were performed using R software (version 4.3.1; R Core Team, 2023) with the “vegan” package (Oksanen et al., 2022).

## 3 Results

### 3.1 Vegetation composition

#### 3.1.1 Coniferous forest (JH1 – JH9)

**Figure 2:**
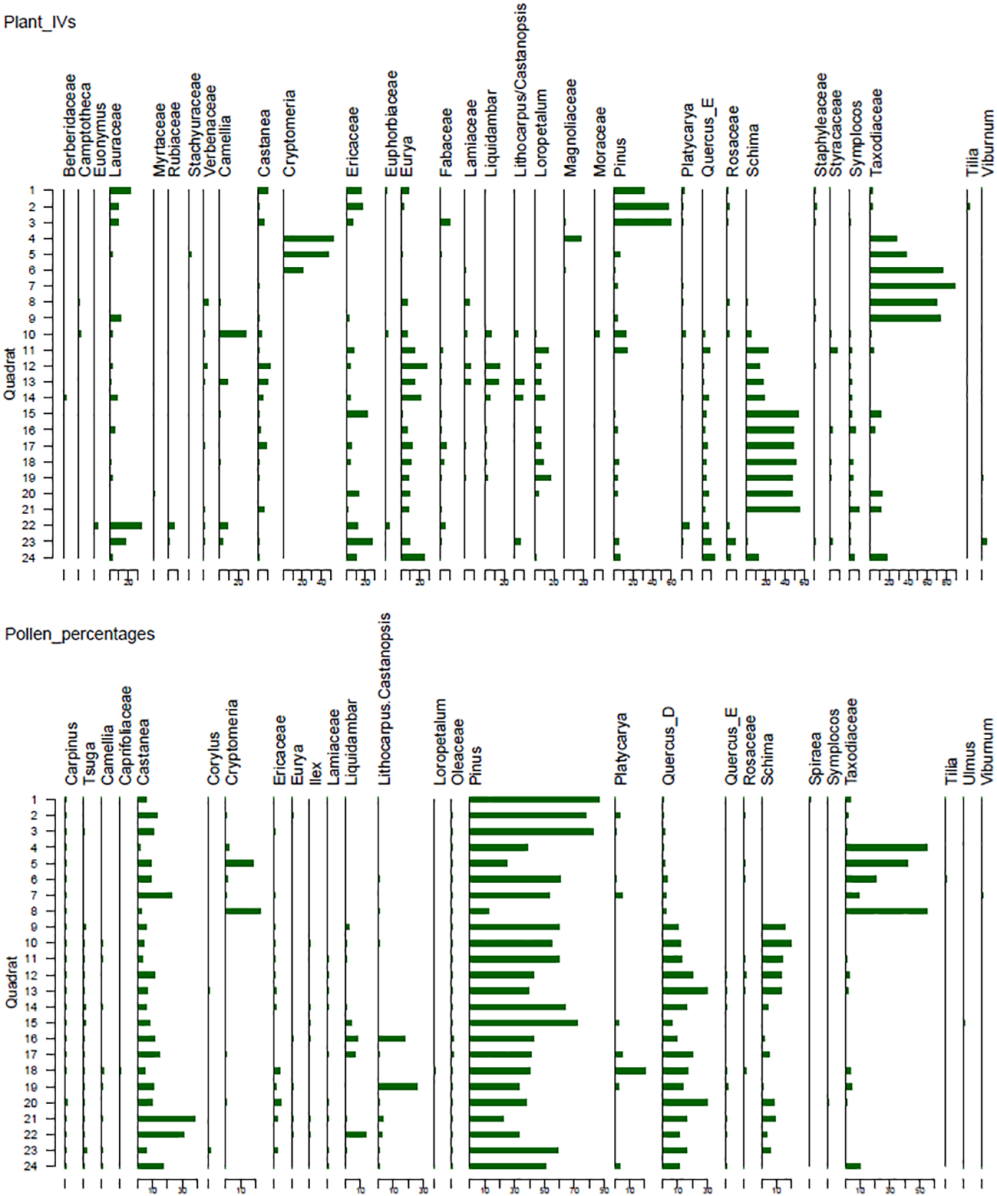
Stratigraphic diagram showing plant IVs and pollen percentages of selected taxa. Taxa with max IVs above 2 and max pollen percentage above 0.5% in at least one quadrat were included.

The *Pin. taiwanensis* forest (JH1 – JH3), situated at elevations above 1000 m, exhibited distinct community stratification (**Figure 2**). The tree layer is predominantly composed of *Pin. taiwanensis*, with a canopy density ranging from 65% to 80%. The shrub layer boasts a coverage of 50–60% and is mainly characterized by species such as *Rhododendron*, *Eurya japonica* Thunb., *Lindera erythrocarpa* Makino, *Euscaphis japonica* Thunberg, and *Lindera reflexa* Hemsl. Meanwhile, the herbaceous layer exhibits coverage varying from 35% to 90%, with prominent species including *Rubus buergeri* Miquel, *Ru. pacificus* Hance, *Justicia quadrifaria* (Nees) T. Anderson, *Achyranthes bidentata* Blume, and fern taxa belonging to *Dryopteris* Adanson and *Athyrium* Roth.

In the *Cryptomeria japonica* (Thunb. ex L. f.) D. Don forest (JH4 – JH5), which is distributed between 900 and 1000 m in elevation, the dominant plants in the tree layer consist of *Cr. japonica* and *Cu. lanceolata*. The shrub layer, characterized by a coverage ranging from 5% to 40%, is primarily dominated by *Cr. japonica*, whereas *Cu. lanceolata*, *Lin. erythrocarpa*, *E. japonica*, and *Magnolia officinalis* Rehder & E. H. Wilson are dominant. The herbaceous layer is primarily comprised of *Ach. bidentata*, *Rehmannia glutinosa* Gaertner, *Reynoutria japonica* Houtt., *Dryopteris* and *Athyrium*.

The *Cu. lanceolata* forest (JH6-JH9) is situated at elevations ranging from 711 to 1100 m. The tree layer is predominantly characterized by *Cu. lanceolata*, with a canopy density of 85–90%. Shrub coverage varies from 5% to 65% and primarily comprises *Lindera glauca* Siebold and Zuccarini, *Lin. reflexa*, *Rhododendron*, *Hydrangea strigosa* Rehder, *Camellia oleifera* Abel, and *Castanea seguinii*. The herbaceous layer, covering 60–90% of the area, is primarily composed of *Rungia densiflora* H. S. Lo, *J. quadrifaria*, *Ach. bidentata*, *Viola arcuata* Blume, *Athyrium*, and *Diplazium donianum* (Mett.) Tardieu.

#### 3.1.2 Mixed forest (JH10 – JH14)

The evergreen deciduous coniferous broad-leaved mixed forest (JH10) is characterized by a community of *Pin. massoniana* and *Liq. formosana*. The canopy density of the tree layer is 80%. In addition to *Pin. massoniana* and *Liq. formosana*, taxa such as *Platycarya strobilacea* Siebold & Zucc., *Castanea henryi*, and *Camptotheca acuminata* Decne. are also abundant. The coverage of shrub layer is 70%, dominated by *Came. oleifera*, *E. muricata*, *Maclura tricuspidata* Carrière and *Lin. glauca*. Vine plants, such as *Trachelospermum jasminoides* (Lindl.) Lem., *Wisteria sinensis* (Sims) Sweet, *Gynostemma pentaphyllum* (Thunb.) Makino, and *Rosa cymosa* Tratt. can also be observed. The herbaceous layer mainly comprises *Liriope spicata* (Thunb.) Lour., and *Athyrium filix-femina* (L.) Roth, with a coverage of 35%.

The evergreen coniferous broad-leaved mixed forest (JH11), dominated by *Sch. superba* and *Pin. massoniana*, primarily occurs at an altitude of approximately 567 m. The tree layer boasts a canopy density of 90%, primarily consisting of *Sch. superba*, *Pin. massoniana*, *Quercus pubescens* Willd., *Loropetalum chinense* (R. Br.) Oliv., and *Styrax confuses* Hemsl. The shrub layer, with a coverage of 65%, comprising *Eur. muricata*, *Sch. superba*, *Lor. chinense*, *Rh. simsii*, *Symplocos sumuntia* Buch.-Ham. ex D. Don, and *Lyonia ovalifolia* (Wall.) Drude. The herbaceous layer, with modest coverage of 7%, occasionally features bamboo and ferns.

The evergreen deciduous broad-leaved mixed forest (JH12 – JH14) spans an altitude range of 550-658 m on Jinhua Mountain. The dominant communities are the *Sch. superba–Liq. formosana–Castanea henryi* forest and the *Sch. superba–Quercus serrata* Thunb.–*Liq. formosana* forest. The tree layer comprises mainly the *Sch. superba*, *Liq. formosana*, *Castanea henryi*, *Q. serrata*, with canopy density of 85 - 90%. The shrub layer, with coverage ranging from 50% to 80%, is mainly composed of *Eur. muricata*, *Sch. superba*, *Lor. chinense*, *Callicarpa bodinieri* H. Lév., *Rhododendron ovatum* (Lindl.) Planch. ex Maxim., and *Clerodendrum cyrtophyllum* Turcz. Vine plants consist of *Gy. pentaphyllum* and *Rosa cymosa*. The herbaceous layer, with coverage varying from 7% to 35%, primarily comprises *Lir. spicata*, *Carex* L., *Pteris* L., and *Dryopteris*.

### 3.1.3 Evergreen broad-leaved forest (JH15 – JH21)

The predominant community within the evergreen broad-leaved forest of Jinhua Mountain is *Sch. superba*, primarily distributed within an altitude range of 520–644 m. The tree layer exhibited a canopy density ranging from 85% to 90%, prominently featuring *Sch. superba* as the dominant and indicator species. It was complemented by *Cu. lanceolata*, *Q. serrata*, *Castanea henryi*, and *Dal. hupeana.* The shrub layer, with coverage varying from 45% to 60%, is mainly composed of *Sch. superba*, *Lor. chinense*, *Eur. muricata*, *Rh. ovatum*, *Albizia kalkora* (Roxb.) Prain, *Symplocos anomala* Brand, *Sy. sumuntia*, and *Quercus glauca* Thunb. The herbaceous layer exhibits coverage ranging from 7% to 30% and is mainly characterized by *Polygonatum odoratum* (Mill.) Druce, *Carex*, *Pteris*, and *Dryopteris*.

### 3.1.4 Shrubland (JH22 – JH24)

The shrubland primarily consists of *Lin. glauca* deciduous shrub (JH21), *Rh. ovatum*–*Lin. glauca–Rh. simsii* shrub (JH23), *Q. serrata*, *Eurya rubiginosa* Hung T. Chang shrub (JH24). The canopy density in these shrublands varied from 45% to 90%. The dominant taxa include *Lin. glauca, Rh. ovatum, Rh. simsii, Q. serrata, Eur. rubiginosa*, *Serissa serissoides* (DC.) Druce, and *Euonymus carnosus* Hemsl. Vine plants, such as *W. sinensis, Actinidia chinensis* Planch., and *Celastrus gemmatus* Loes. can be found in these shrublands. The coverages of the herbaceous layer range from 2% to 30%.

### 3.2 Pollen composition

A total of 109 pollen taxa have been identified in the soil samples, with the majority belonging to the arboreal taxa (**Figure 2**). The tree taxa primarily include *Pinus* L., Taxodiaceae Warming, *Cryptomeria* D. Don, *Tsuga* (Endl.) Carrière, *Schima* Reinw. ex Blume, *Castanea* Mill., *Liquidambar* L., deciduous *Quercus* (*Quercus*_D), *Platycarya*, evergreen *Quercus* (*Quercus*_E), *Lithocarpus* Blume/*Castanopsis* Spach, *Betula* L., *Corylus* L., and Euphorbiaceae Juss. There are also 23 shrub taxa, mainly composed of Ericaceae Juss., Caprifoliaceae Juss., *Viburnum* L., Rosaceae Juss., *Ilex* L., *Camellia* L., *Eurya* Thunb., Moraceae Gaudich., and Lamiaceae Martinov. The herbaceous taxa include Asteraceae Bercht. & J. Presl, *Artemisia* L., Polygonaceae Juss., Brassicaceae Burnett, Poaceae Barnhart, Acanthaceae Juss., and Saxifragaceae Juss. Fern taxa are abundant, with frequent occurrences of Polypodiaceae J. Presl & C. Presl, *Dryopteris*, *Hicriopteris* Presl/*Dicranopteris* Bernh., *Pteris* and *Lygodium* Sw.

#### 3.2.1 Coniferous forest (JH1 – JH9)

The *Pin. taiwanensis* forest (JH1 – JH3) is characterized by a substantial content of coniferous pollen taxa, notably *Pinus* (82.1%) and Taxodiaceae (2.3%). Broad-leaved taxa are mainly composedprimarily consisted of *Castanea* (10%), *Platycarya* (1.6%), *Quercus*_D (1%), and *Eurya* (0.5%). The shrub taxa can be found of *Rhododendron* and Ericaceae. The content of herbaceous taxa is low, mainly including *Artemisa*, Asteraceae, and Saxifragaceae. Fern taxa comprise mainly *Hicriopteris*/*Dicranopteris* and Polypodiaceae.

In the *Cr. japonica* forest (JH4 – JH5), there are elevated contents of Taxodiaceae (48.6%), *Pinus* (31.6%), and *Cryptomeria* (10.2%). Deciduous arboreal taxa mainly include *Castanea* (5.4%), *Quercus*_D (0.9%), *Corylus*, and *Platycarya*. The content of evergreen broad-leaved taxa is limited, mainly comprising *Eurya* and *Quercus*_E. The shrub taxa consist of Rosaceae (0.6%), Caprifoliaceae, and *Salix.* Herbaceous taxa are composed mainly of *Artemisa* (1.9%) and *Aster* (0.5%). *Hicriopteris*/*Dicranopteris* (2.9%) and Polypodiaceae (2.7%) are abundant.

Within the *Cu. lanceolata* forest (JH6 -JH9), there are notable contents of *Pinus* (46.8%), Taxodiaceae (21.2%), and *Cryptomeria* (6.1%). Broad-leaved taxa mainly comprise *Castanea* (10.1%), *Quercus*_D (4.5%), *Schima* (4.1%), *Platycarya* (1.6%), *Liquidambar* (0.6), *Lithocarpus*/*Castanopsis*, *Betula*, and *Corylus*. Herbaceous taxa mainly include *Artemisia* (3.5%), Asteraceae (1.1%), and *Aster* L. (0.5%). The fern taxa are mainly composed of *Hicriopteris*/*Dicranopteris* (9.6%), Polypodiaceae (9.2%), and *Athyrium* (1.6%).

#### 3.2.3 Mixed forest (JH10 – JH14)

In the evergreen deciduous coniferous broad-leaved mixed forest (J10), there is a notable presence of coniferous pollen taxa including *Pinus* (54.6%), *Tsuga* (0.7%), and *Taxodiaceae* (0.6%). Broad-leaved trees and shrub taxa mainly comprise *Schima* (20.1%), *Quercus*_D (12%), *Castanea* (4.4%), Ericaceae (1.2%), *Liquidambar* (0.9%), *Camellia* (0.8%), and *Corylus*, *Platycarya*, *Ulmus*, and *Eurya*. The herbaceous taxa mainly comprise *Artemisia* (1.1%). Fern taxa includes Polypodiaceae (27.2%), *Hicriopteris/Dicranopteris* (13.3%), and *Dryopteris* (5.9%).

In the evergreen coniferous broad-leaved mixed forest (JH11), high contents of *Pinus* (60.1%), *Schima* (14.5%), and *Quercus*_D (13.2%) are prevalent. Taxa, such as *Castanea* (3.1%), *Liquidambar* (1.1%), *Rhododendron* (1.1%), Taxodiaceae (0.9%), *Camellia* (0.8%), *Tsuga* (0.8%), Rosaceae (0.7%), *Corylus* (0.6%), and Labiatae (0.5%) are also abundant. Herbaceous taxa mainly comprise *Artemisia* (1.5%), whereas fern taxa is mainly composed of *Hicriopteris*/*Dicranopteris* (8.3%).

Within the evergreen deciduous broad-leaved mixed forest (JH12 – JH14), the contents of arboreal pollen are high, including *Pinus* (48.8%), *Quercus*_D (22.1%), *Schima* (10.4%), *Castanea* (8.3%), Taxodiaceae (1.9%), Ericaceae (1.5%), Lamiaceae (0.9%), *Tsuga* (0.9%), Rosaceae (0.9%), *Quercus*_E (0.7%), *Corylus* (0.6%) and *Liquidambar* (0.5%). Herbaceous taxa are low, mainly composed of *Artemisia* (1.6%) and Asteraceae. The content of fern taxa is high, with Polypodiaceae of 16.3% and *Hicriopteris*/*Dicranopteris* of 16.2%.

#### 3.2.4 Evergreen broad-leaved forest (JH15 – JH21)

Pollen composition mainly comprises *Pinus* (41.5%), *Quercus*_D (16.1%) and *Castanea* (14.2%). The evergreen components, including *Lithocarpus*/*Castanopsis* (6.9%), *Platycarya* (4.5%), and *Schima* (3.9%), are substantial. Additionally, there were abundant taxa, such as *Liquidambar* (2.9%), Ericaceae (2%), Taxodiaceae (1.6%), *Tsuga* (0.8%), *Camellia* (0.6%), *Quercus*_E (0.6%), *Eurya* (0.5%), and Oleaceae (0.5%). Herbaceous taxa mainly include *Artemisa* (1.5%), whereas fern taxa comprise mainly *Hicriopteris*/*Dicranopteris* (17.8%).

#### 3.2.5 Shrubland (JH22 – JH24)

The pollen assemblage is characterized by high contents of *Pinus* (47.8%), *Castanea* (18%), *Quercus*_D (12.8%), *Liquidambar* (4.8%), Taxodiaceae (3.7%), *Schima* (3.3%), *Platycarya* (1.3%), Ericaceae (1.2%), *Lithocarpus/Castanopsis* (1%), *Corylus* (0.7%), Lamiaceae (0.6%), *Quercus*_E (0.6%), *Camellia* (0.5%), and *Eurya* (0.5%). *Artemisa* (2.7%) and Asteraceae (0.9%) are abundant. The fern taxa mainly comprise *Hicriopteris*/*Dicranopteris* (20.5%) and Polypodiaceae (3%).

### 3.3 Correlation between vegetation and pollen

**Figure 3:**
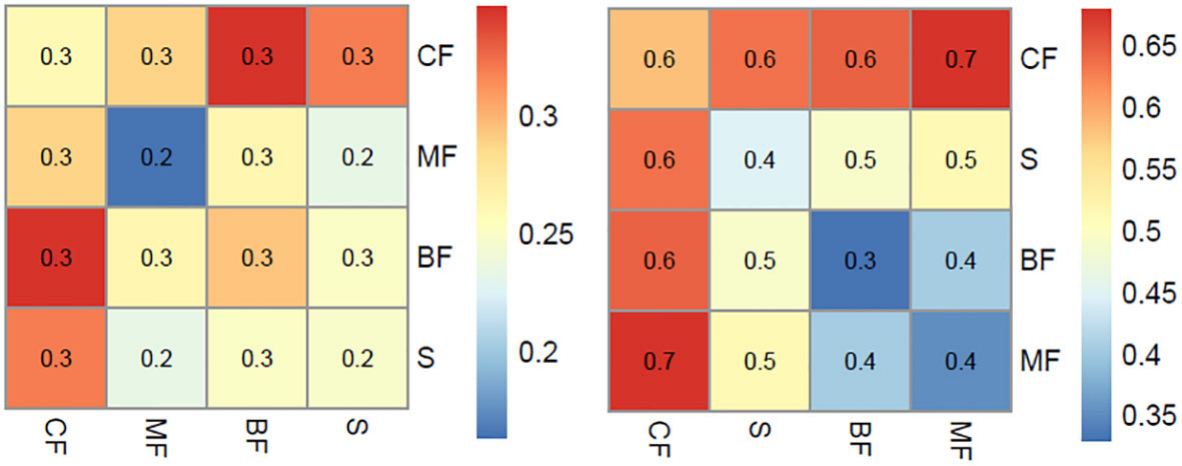
Mean distance within same and among different vegetation types for pollen (left) and vegetation (right).

MRPP analysis revealed significant differences in both plant IVs and pollen signals among the various vegetation types (*p* < 0.001). In general, the compositional differences among plants within the same community are smaller than those observed among different vegetation types (**Figure 3**). This trend is also reflected in the differences in pollen composition. However, it is noteworthy that the compositional distance in the pollen analysis is notably smaller than that observed in the plant composition. For example, the compositional distance between coniferous forests and other communities typically exceeds 0.6 in quadrat data, contrasting with a distance of 0.3 in pollen analysis. Similarly, the compositional difference between broad-leaved forest and broad-leaved and coniferous mixed forest is low in both plant and pollen results, with mean distances of 0.3 and 0.3-0.6, respectively.

**Figure 4:**
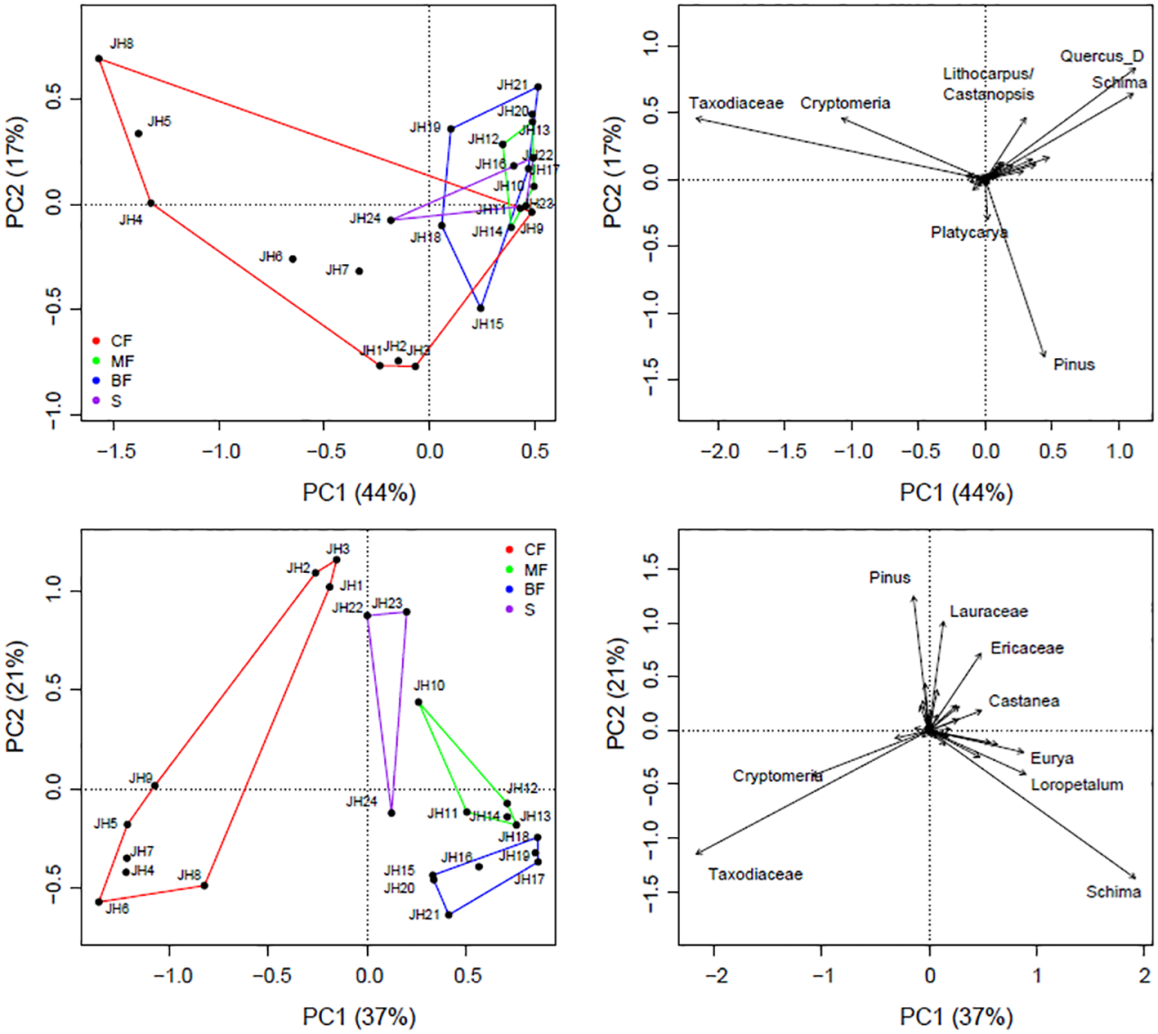
PCA results of pollen (upper) and vegetation (lower) composition from 24 quadrats of Jinhua Mountain (JH1-24). Only selected taxa labeled.

Ordination analysis effectively segregated quadrats with different vegetation types. Both plant IVs and pollen signals can be differentiated based on their compositional differences, with the first axes explaining approximately 44% (IVs) and 37% (pollen) of the variations, respectively. Coniferous forests are generally characterized by high contents of *Pinus*, Taxodiaceae, and *Cryptomeria*, which are distinctly different from those in broad-leaved forests (**Figure 4**).

**Figure 5:**
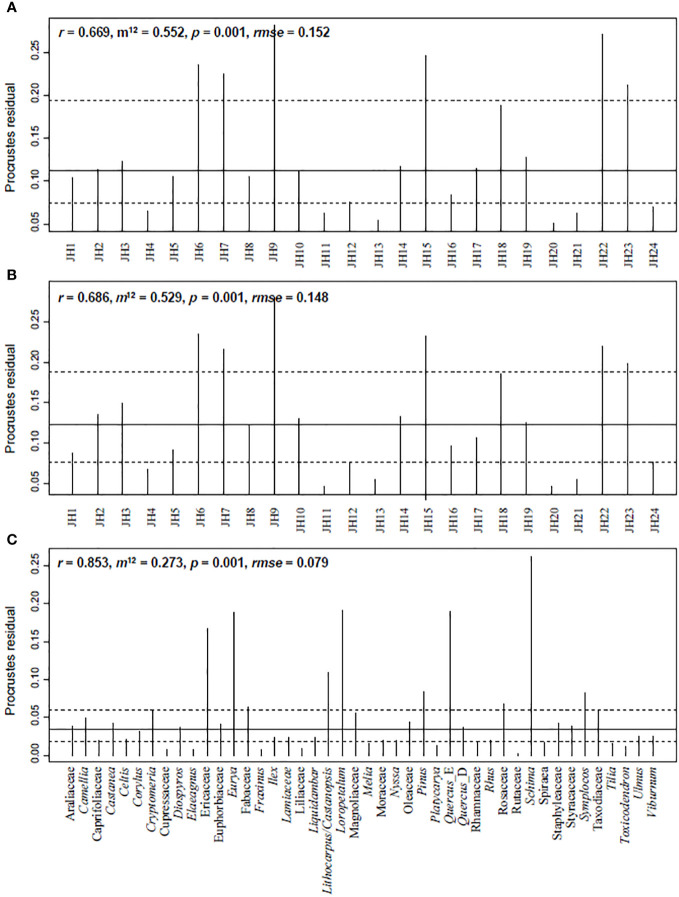
Procrustes residual results illustrating the similarity between plant IVs and pollen signals from quadrat data in Jinhua Mountain. **(A)** Based on all the plant and pollen taxa; **(B, C)** Based on 44 shared taxa between plant and pollen. *r*, correlation in a symmetric Procrustes rotation; *m* ^12^, Procrustes sum of squares; *rmse*, Procrustes root mean squared error.

The procrustes and protest results indicate a significant similarity between plant IVs and pollen percentages (**Figure 5**). Correlations in symmetric Procrustes rotation are 0.67 at the site level, considering all pollen and plant taxa. When the analysis is conducted with the 44 shared taxa between plants and pollen, the correlations in symmetric Procrustes rotation increase to 0.69 at the site level and 0.85 at the taxa level, respectively. Quadrats of JH6, JH7, JH9, JH15, and JH22 are with high residuals, as well as taxa of *Schima*, *Loropetalum*, *Quercus*_E, *Eurya*, and Ericaceae.

## 4 Discussion

Pollen analysis is a significant botanical and ecological tool for studying the dynamics of biota and biotic systems (Prentice et al., 1998; Seddon et al., 2014; Kong et al., 2018; Birks, 2019). By comparing pollen analysis with quadrat investigations, our results unequivocally demonstrated that pollen signals can accurately reflect the major plant community composition and emphasize the significance of specific plant taxa. Consequently, pollen analysis in subtropical forests holds ecological significance as it bridges the gap between current and paleoecology to some extent, bolstering our confidence in studying long-term plant community and vegetation composition changes using fossil pollen in subtropical forests.

In agreement with previous pollen analyses in subtropical China (Liu et al., 2001; Yu et al., 2002; Zheng et al., 2002; Zheng et al., 2007; Zheng et al., 2008; Ding et al., 2011; Fang et al., 2015; Wan et al., 2020), pollen composition in surface soil from Jinhua Mountain closely aligns with modern vegetation, indicating that pollen signals vary according to different plant communities. The landscape of subtropical China is characterized by moderate mountainous and hilly terrain, where mountain vegetation exhibits distinct variations along altitude gradients. On Jinhua Mountain, both vegetation investigations and pollen analyses reveal vertical variations in plant composition signals. In summary, a higher prevalence of evergreen broad-leaved taxa has been observed in the lower regions, whereas cold-tolerant coniferous taxa tend to be dominated at higher elevations (**Figure 2**). This phenomenon corresponds to pollen analyses conducted on Tianmu Mountain in Zhejiang Province (Guo, 2021), Yushan Mountain in Taiwan (Yu et al., 2002), Xishan Mountain in Jiangxi Province (Li et al., 2016), and Shennongjia Mountain in Hubei Province (Liu et al., 2001). Pollen composition in surface soil effectively reflects the different vegetation communities, such as pine forests and *Schima* forests, and their geographic distribution closely mirrors that of actual vegetation. The prevalence of *Pinus* pollen, particularly in pine forests, is evident in nearly all the samples. Considering that *Pin. taiwanness* forest is situated at higher altitudes and that *Pin. massoniana* can also be found sporadically in other plant communities on Jinhua Mountain (Fan et al., 2019), the high presence of *Pinus* pollen in our study region is justified. This finding is consistent with previous researches indicating that a high proportion of *Pinus* pollen is indicative of pine forests or isolated occurrence within the study region (Li and Yao, 1990; Xu et al., 2007; Xiao et al., 2011). In contrast to *Pinus* pollen, a high content of Taxodiaceae pollen is primarily associated with the cedar forests (**Figure 2**). This may be attributed to both low pollen productivity and the limited dispersal ability of *Cu. lanceolata* within modern vegetation (Xiao et al., 2011; Li et al., 2018).

Furthermore, our results illustrate that pollen percentage data aligns with the IVs derived from quadrat-based surveys of different plant communities. Pollen percentages provide quantitative information about pollen composition, enabling complex numerical analyses to reveal potential dynamics and driving forces. Therefore, this method is the most convenient and widely applied approach for pollen analysis. In synecology, IV serves as a comprehensive quantitative indicator of a given taxon’s status and role in the community (Song, 2016), synthesizing relative density, dominance, and frequency (Curtis and Mclntosh, 1951). Owing to its simplicity and clarity, IV has been a common tool in community ecology for many years (Song, 2016). According to the protest results, there is a significant correlation between the pollen percentages and IVs (**Figure 5**). This correlation is reasonable because dominant and abundant taxa are more likely to produce a greater number of pollen grains than rare taxa within the community (Andersen, 1970). For example, *Cr. japonica*, a dominant and edificator species in JH4-JH5, corresponds well with the high *Cryptomeria* pollen percentage in the soil samples. Similarly, high IVs for *Cu. lanceolata*, *Castanea*, and *Liquidambar* are consistent with the elevated pollen percentages of Taxodiaceae, *Castanea*, and *Liquidambar* in the same quadrats. This suggests that the commonly used pollen percentage inherently preserves the quantitative characteristics of the source plants and can effectively reflect the importance of dominant taxa in the plant community.

**Figure 6:**
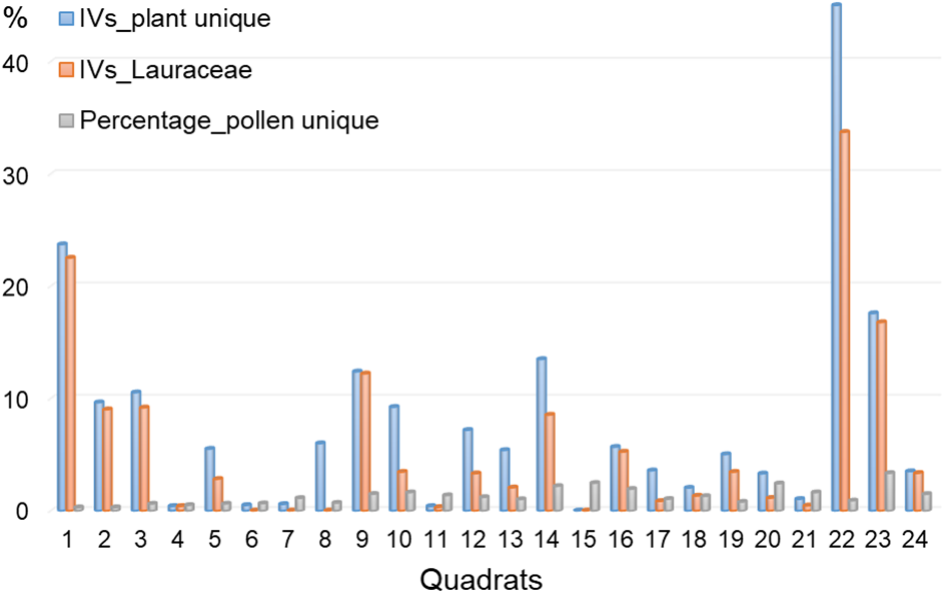
Unique plant IVs and pollen percentages in different quadrats revealing overall taxa mismatch between two data sets.

It is essential to note the discrepancies between pollen composition and plant signals in our study, including overall compositional levels and individual numeric features (**Figures 2**, **5**, **6**). Our results revealed that plant taxa do not precisely align with pollen composition. This discrepancy is particularly evident for certain rare taxa such as *Alangium* Lam., Rubiaceae Juss., and Verbenaceae J. St.-Hil. Conversely, some pollen taxa, including *Alnus*, *Salix*, and *Pterocarya*, were absent in the plant investigation. We propose that these mismatches stem from the limitations of the quadrat area and dispersal ability of pollen (Andersen, 1970; Jackson and Kearsley, 1998). In subtropical China, the *RSAP* is estimated to range from approximately 290-500 m (Chen, 2019; Jiang et al., 2020; Guo, 2021). Consequently, a forest quadrat area of 30 × 30 m may not be sufficient to capture all existing taxa (Fan et al., 2019). Concerning individual numeric features, pollen percentages do not always align with the IVs for specific taxa. The taxa absent in the pollen data exhibited an average IV of 8, contrasting with an average percentage of 1.8% for unique pollen taxa not recorded in the plant investigation (**Figure 6**). Strikingly, Lauraceae Juss. possesses an average IV of 5.8 in 24 quadrats, yet its pollen is absent in the quadrat soils (**Figure 6**). Lauraceae is common in tropical and subtropical forests (Tan et al., 2023), and many plants in this family are insect-pollinated. In our dataset, the Lauraceae plants mainly comprise *Lin. glauca*, *Lin. erythrocarpa*, and *Lin. Reflexa*, all of which are insect-pollinated characterized by small, fragrant flowers with floral glands. Similarly, *Sch. superba* is a dominant species and edificator in evergreen broad-leaved forests on Jinhua Mountain, but its pollen percentage does not align with IVs (**Figure 5**). Like Lauraceae, *Sch. superba* is a xenogamous plant that is highly depended on insect pollinators for seed production (Bai et al., 2022). In contrast to Lauraceae and *Schima* pollen, *Pinus* is the dominant species in pine forests, yet its pollen can be found high percentages in nearly all samples. These observations underscore another critical issue: the different pollen dispersal strategies of plants might affect the relationship between pollen percentages and plant IVs (Ackerman, 2000). In subtropical and tropical forests, the high frequency of insect-pollinated taxa (Wang et al., 2023) can introduce substantial uncertainties in the relationship between pollen and vegetation, leading to the underestimation of insect-pollinated taxa, such as Lauraceae, in pollen analysis.

The application of recently published *RPP* and *R* values to pollen percentage data yielded limited improvements in our results. Fossil pollen grains preserved in soil and other archives are subject to varying pollen productivity, transportation processes, and preservation conditions, resulting in nonlinear relationships between pollen and vegetation, and uncertainties in pollen percentages when representing the importance of selected taxa (Sugita, 1994; Bunting et al., 2004). *RPP* estimations for selected taxa have been reported (Li et al., 2018; Wieczorek and Herzschuh, 2020), as have regional variations in pollen *R* values in China (Xia and Ni, 2024). We checked the *RPP* results from tropical and subtropical forests (Chen, 2019; Jiang et al., 2020; Guo, 2021). We found that applying these *RPP*s to *Pinus*, *Castanea*, *Castanopsis*/*Lithocarpus*, and Moraceae pollen percentage data led to changes and introduced additional biases. Given that pollen-plant abundance data were collected from regions with different forest compositions, this calibration should be avoided (Sugita, 1994). A similar situation arises with *R* values in China, which exhibit significant regional variability (Xia and Ni, 2024). As a result, we refrained from this calibration and focused on the raw pollen percentages. Nevertheless, our results indicated a significant consistency between pollen percentages and plant IVs (**Figure 5**), which may hold significance for pollen analysis across different vegetation types within a small region.

Despite the inherent uncertainties, the consistency between pollen percentages and plant IVs in subtropical forests from Jinhua Mountain is superior to that observed in open landscapes from the Northeast China Transect studied by Tan (2020). We propose that the main reason for this disparity lies in the different *RPP* and *RSAP* values of the two regions. For instance, *Artemisia* and Amaranthaceae Juss. (Chenopodiaceae) are indicative pollen taxa for typical grasslands in northern China, characterized by high *RPP*s and *RSAP*s that can extend over 1000 km (Xu et al., 2016; Cao et al., 2017). The influx of long-distance pollen into local pollen rain undoubtedly introduces bias into the relationship between pollen percentages and plant IVs Tan (2020). In the current study, the plant investigation data did not include herbaceous and fern species because of their limited coverage and biomass. The average percentages of herbaceous pollen and ferns in the pollen data are 0.1% and 1.6%, respectively. Therefore, we propose that our data exhibits regional representativeness for subtropical forests. Consequently, pollen analysis based on lake or mountain peat in subtropical China, which often maintains a high percentage of herbaceous pollen, may have underscored the development of local grasslands/wetlands while underestimating forest coverage.

In summary, our results demonstrated a significant correlation between pollen percentages and plant IVs, reinforcing the utility of pollen analysis in reflecting the ecological importance of specific taxa within a plant community. Discrepancies between pollen composition and plant signals exist, which probably stem from the limitations of the quadrat area and pollen dispersal ability. Despite inherent uncertainties, the consistency between pollen percentages and plant IVs in subtropical forests from Jinhua Mountain is superior to that observed in open landscapes. These relationships may also hold promise in paleoecology, where pollen analysis can be combined with geochemical and molecular information from natural archives to reconstruct ecological and evolutionary systems deep into the past.

## Data availability statement

The original contributions presented in the study are included in the article/**Supplementary Material**. Further inquiries can be directed to the corresponding author.

## Author contributions

KL: Formal analysis, Funding acquisition, Methodology, Writing – original draft, Writing – review & editing. BT: Investigation, Methodology, Writing – review & editing. ML: Writing – review & editing, Methodology. JN: Conceptualization, Funding acquisition, Supervision, Writing – review & editing.
